# From stroke to depression: The need for systematic screening for post-stroke depression

**DOI:** 10.4102/sajpsychiatry.v30i0.2346

**Published:** 2024-12-16

**Authors:** Mundih N. Njohjam, Swirri S. Nji, Ebsiy M. Nongse

**Affiliations:** 1 Department of Neurology, Faculty of Medicine, Pharmacy and Odonto-stomatology, Cheikh Anta Diop University, Dakar, Senegal; 2 Department of Public Health, Faculty of Heath Sciences, University of Bamenda, Bamenda, Cameroon; 3 Department of Internal Medicine, Cameroon Baptist Convention Health Services, Bamenda, Cameroon

**Keywords:** stroke, post-stroke depression, prevalence, mental health, stroke survivors

## Abstract

**Background:**

Post-stroke depression (PSD) negatively impacts the physical and mental well-being of stroke survivors. However, data on the prevalence and risk factors of PSD in African countries such as Cameroon are limited.

**Aim:**

This study aims to determine the prevalence and factors associated with PSD among stroke survivors at a hospital in Cameroon and inform clinical practice.

**Setting:**

The study was carried out in the Nkwen Baptist Hospital in the North West region of Cameroon.

**Methods:**

This was a hospital-based cross-sectional study. Stroke patients were systematically screened for PSD using the patient health questionnaire (PHQ-9). PSD was present if a patient scored ≥ 4 points on the scale. The multidimensional scale of perceived social support was used to assess the level of social support, the modified Rankin tool and Barthel index were used to assess functional independence, and the Fatigue assessment tool was used to assess post-stroke fatigue. A multivariate analysis was performed to identify factors associated with PSD.

**Results:**

A total of 103 patients were included in the study. The mean age was 55.55 ± 12.15. Most patients were males (58.25%). The mean depression score was 5.17 ± 6.26. The overall prevalence of PSD was 36.89%. A higher functional impairment, post-stroke fatigue, perceived social support, recent stroke and being divorced were all associated with high PSD scores.

**Conclusion:**

In this study, we found a high prevalence of PSD using a systematic screening approach, suggesting that systematic screening for PSD can lead to early detection and management.

**Contribution:**

Systematic screening for PSD in stroke patients can lead to early diagnosis and, consequently, early initiation of treatment. Integration of mental health support and care as part of the routine stroke is warranted.

## Introduction

Stroke is the leading cause of acquired disability globally.^1^ For many stroke survivors, stroke-associated disability is only one of several debilitating sequelae they must overcome. Psychological comorbidities, such as post-stroke depression (PSD), are common in life after a stroke and can negatively affect the quality of life, delay recovery and hinder social reintegration.^2,3,4^ Post-stroke depression is the most prevalent mental health disorder following a stroke.^5^ Current data on PSD in sub-Saharan Africa (SSA) suggest that approximately 1 in 3 individuals who have had a stroke experience clinical depression.^6^ Similarly, a study on PSD in Ghana indicated that nearly 4 out of every 10 stroke survivors exhibit clinically significant depression.^7^

Post-stroke depression increases the risk of subsequent strokes.^5^ A meta-analysis aimed at determining the risk of recurrent stroke among stroke survivors with PSD suggested that PSD may be an independent predictor of stroke recurrence in ischaemic stroke patients, with a pooled adjusted relative risk for stroke recurrence of 1.48.^5^ Additionally, PSD is associated with increased mortality at 12 and 24 months post-stroke.^8^ While PSD has been extensively researched in developed countries, various clinical and psychosocial risk factors have been identified, including age, cognitive impairment, previous history of depression, stroke severity and lack of social support.^9,10,11,12^ However, data on PSD in SSA are limited, with only a few studies conducted, primarily in West Africa.^6,7,13,14^ The critical shortage of psychiatrists and mental health specialists across the African continent hampers the optimal treatment of PSD, as many stroke care guidelines may not effectively incorporate mental health support for stroke survivors.^15^

Effective management of PSD requires early detection and the initiation of treatment.^16^ However, PSD often goes unrecognised and undiagnosed in a significant number of stroke survivors, leading to delays in treatment.^16^ Patients are typically screened for PSD only if they or their caregivers report symptoms. The aim of this study was to determine the prevalence and factors associated with post-stroke depression among stroke survivors at a hospital in Cameroon and to inform clinical practice through a systematic screening approach.

## Research methods and design

### Study design and settings

This was a cross-sectional hospital-based study conducted at the Nkwen Baptist Hospital in the North West region of Cameroon from March 2023 to March 2024. Patients were recruited from both the outpatient and inpatient units of the Department of Internal Medicine. The Nkwen Baptist Hospital is one of three referral hospitals in the North West region of Cameroon, with a daily patient turnover of over 600 clients and approximately 18 000 patients monthly.^17^

### Participants

Our study population included stroke survivors who were either outpatients attending follow-up visits or inpatients admitted for stroke treatment. We used a consecutive sampling method, including participants who willingly provided informed consent to participate in the study.

### Inclusion criteria

The participant must be at least 18 years old and diagnosed with a stroke. The participant should provide informed consent and be able to read, understand and complete the questionnaires.

### Exclusion criteria

Participants who do not provide informed consent and participants who are unable to read, understand or complete questionnaires.

### Data collection tools

To collect demographic and clinical data, including age, sex, level of education, occupation, marital status, comorbidities, time since stroke, stroke type and physical examination findings, a pre-established, pre-tested and validated questionnaire was utilised. All eligible stroke survivors presenting at the study site were systematically screened for PSD, regardless of whether they or their caregivers reported any symptoms of depression.

### Patient Health Questionnaire

To assess depression, we used the Patient Health Questionnaire (PHQ-9) (see Online Appendix 1) scale. The PHQ-9 is a 9-item screening tool designed to diagnose and gauge the severity of depression. It is adapted from the Diagnostic and Statistical Manual of Mental Disorders, 4th edition (DSM-IV) and includes the same diagnostic symptom criteria outlined in the DSM-IV.^18^ Respondents are asked to recall and rate their experiences with depressive symptoms over the past 2 weeks. Each item on the scale is evaluated on a severity scale ranging from 0 to 3.^19^ The minimum score is 0 and the maximum score is 27. A score of 5 or higher is considered indicative of clinical depression. The scores are further categorised into minimal, mild, moderate, moderately severe and severe depression as follows: 1–4: minimal depression; 5–9: mild depression; 10–14: moderate depression; 15–19: moderately severe depression and 20–27: severe depression.

The PHQ-9 has been validated for PSD screening in different populations.^20^

### Barthel index

The Barthel index for activities of daily living was used to assess functional impairment in performing daily activities.^21^ This tool is widely utilised in stroke care and research to evaluate functional independence among stroke survivors. It assesses 10 common activities of daily living, including feeding, bathing, grooming, dressing, bladder and bowel control, toilet use and mobility on flat surfaces. Additionally, it has been adapted and culturally validated in various settings for patients with neurological diseases, including stroke.^22,23^

### Modified Rankin score

Modified Rankin score (MRS) is a single-item global outcome tool that has been validated and is widely used in clinical practice and research.^24,25^ It is used to assess the level of functional independence based on pre-stroke activities.

### The Multidimensional Scale of Perceived Social Support

The Multidimensional Scale of Perceived Social Support (MSPSS)^26^ is a 12-item tool commonly used to assess a respondent’s perception of the adequacy of social support from three sources: family, friends and a significant other. Responses are graded based on 5-point Likert scale (0 = strongly disagree, 5 = strongly agree).

### Fatigue Assessment Scale

Fatigue Assessment Scale (FAS) is a 10-item self-reported scale that evaluates symptoms of chronic fatigue.^27^ It assesses both physical and mental fatigue. Each item is rated on a 5-point Likert scale ranging from 1 (‘never’) to 5 (‘always’). The total score can range from 10 to 50, with higher scores indicating greater fatigue. It is one of the most widely used tools for the assessment of post-stroke fatigue.^28^

### Data analysis

Data collected were entered and analysed using Statistical Package for Social Sciences (SPSS) version 26. Descriptive statistics including frequencies, means and medians were generated for different variables. The level of statistical significance was set at *p* < 0.05. Univariate and multivariate logistic regression analyses were performed to identify factors associated with depression.

### Ethical considerations

Ethical clearance for this study was obtained from the institutional ethical clearance review board of the Cameroon Baptist Convention Health Services (No. IRB2023-06). This study was part of a bigger study to determine the aetiologies and other clinical aspects of stroke. Each patient was duly informed of the study objectives, procedures and any risks associated with the study, and informed consent was obtained from all study participants prior to inclusion in the study. Participants were told they had the right to withdraw from the study at any point without any justification. Data from each participant were coded and anonymised to ensure confidentiality. No data that could identify any of the participants were collected. All participants who screened positive for depression were referred to a mental health specialist for management.

## Results

### Sociodemographic characteristics

A total of 103 patients were included in the study. The mean age was 55.55 ± 12.15 with a minimum age of 26 and a maximum age of 70. Most patients were males (58.25%) as against 41.75% for females. Table 1 shows the sociodemographic profile of the participants.

**TABLE 1:** Demographic profile of participants.

Variable	*n*	%
**Sex**		
Male	60	58.25
Female	43	41.75
**Marital status**		
Single	10	9.71
Engaged	21	20.39
Married	66	64.08
Divorced	6	5.83
**Level of education**		
Primary	10	9.71
Secondary	66	64.08
Tertiary	20	19.42
None	7	6.80
**Residence**		
Urban	66	64.08
Rural	37	35.92
Total	103	100.00

### Clinical characteristics

Table 2 summarises the clinical characteristics of the study cohort. Time since stroke was between 1 and 6 months for most participants while ischaemic stroke was the most common type of stroke.

**TABLE 2:** Clinical characteristics.

Variable	Frequency	%
**Time since stroke**		
1–6 months	55	53.40
6–12 months	31	30.10
< 1 month	16	15.53
> 12 months	1	0.97
**Stroke type**		
Ischaemic	66	64.08
Haemorrhagic	37	35.92

### Post-stroke fatigue, perceived social support and functional impairment

Table 3 shows the different mean scores for post-stroke fatigue, perceived social support and functional impairment.

**TABLE 3:** Mean scores for post-stroke fatigue, perceived social support and functional impairment.

Variable	Barthel index score	FAS score-mental	Perceived social support score	FAS-physical
Minimum	20	4	15	4
Maximum	95	15	84	15
Mean ± Std.	67.04 ± 16.00	7.88 ± 2.37	56.47 ± 18.57	7.51 ± 2.38

FAS, Fatigue Assessment Scale.

A significant number of patients had severe functional impairment, with low perceived social support and high post-stroke fatigue scores.

### Post-stroke depression

The mean score for PSD was 5.17 ± 6.26. All participants who screened positive for PSD were further classified based on the severity of symptoms into mild, moderate, moderately severe and severe. Table 4 shows the classification of PSD based on severity.

**TABLE 4:** Classification of post-stroke depression based on severity.

PSD severity	*n*	%
Mild	21	20.39
Moderate	6	5.83
Moderately severe	6	5.83
Severe	5	4.85
Total	38	36.89

Note: There was a high rate of post-stroke depression in our study cohort.

PSD, post-stroke depression.

### Factors associated with post-stroke depression

Table 5 shows the different factors associated with PSD after univariate analysis. A higher functional impairment (low Barthel index score, higher MRS), post-stroke fatigue, low level of perceived social support and being divorced were associated with higher PSD scores.

**TABLE 5:** Factors associated with post-stroke depression.

Variable	*p*	95% confidence interval	
		Lower bound	Upper bound
FAS-mental	0.031	0.05	1.03
FAS-physical	0.004	0.24	1.28
MRS	0.029	−3.41	−0.19
BI	< 0.001	−0.50	−0.33
Marital status: Divorced	0.020	2.51	27.90

BI, Barthel index; FAS, Fatigue Assessment Scale; MRS, modified Rankin score.

### Multivariate

A multiple linear regression analysis was performed to examine the factors that influence PSD scores. Table 6 shows the results of the analysis.

**TABLE 6:** Factors associated with post-stroke depression after multivariable analysis.

Model	*p*	95% confidence interval	
		Lower bound	Upper bound
Age	0.507	−0.07	0.15
Sex	0.389	−1.25	3.17
Level of education: Primary	0.759	−11.89	16.24
Level of education: Secondary	0.635	−8.33	13.57
Level of education: Tertiary	0.915	−12.62	14.06
Residence: Rural	0.172	−0.72	3.97
Marital status: Engaged	0.304	−16.13	5.09
Marital status: Married	0.179	−17.56	3.32
Marital status: Divorced	0.616	−11.29	18.93
Time since stroke: 1–6 months	**0.013**	1.76	14.30
Time since stroke: 6–12 months	**0.025**	0.88	12.92
Time since stroke: > 12 months	0.063	−0.45	17.00
Stroke type: Haemorrhagic	0.676	−2.13	3.27
MRS	0.738	−2.23	3.13
Barthel index score	0.135	−0.27	0.04
Fatigue assessment score-mental	0.800	−0.82	0.63
FAS-physical	0.226	−0.31	1.31
Co-morbidities: Hypertension	0.849	−3.58	2.95
Co-morbidities: Diabetes	0.558	−3.82	2.07
Co-morbidities: Heart disease	0.933	−3.58	3.29
Co-morbidities > 1 co-morbid condition	0.621	−4.52	2.72
Perceived social support score	**0.009**	−0.20	−0.03

Note: Figures in bold shows: After multivariate analysis, Time since stroke 1–6 and 6–12 months as well as perceived social support were the only factors that were significantly associated with post-stroke depression.

FAS, fatigue assessment score; MRS, modified Rankin score.

The prediction model showed a very high positive relationship between the observed values (correlation coefficient = 0.76) and the prediction, which explains 58.18% of the variance in the PSD scores. The ANOVA results showed that our regression model is statistically significant (*p* < 0.001), suggesting a good fit. A shorter duration of stroke and decreased social support were significantly associated with higher PSD scores.

## Discussion

We found a high level of PSD using systematic screening. These results are similar to those reported from other sub-Saharan African countries.^6,14,29,30,31^ This high prevalence of stroke raises several considerations. Initially, this high prevalence is likely linked to the broader challenges of stroke management in SSA. Over the last decade, the incidence and stroke-associated morbidity as well as mortality rates in Cameroon have been rising persistently at an alarming rate, compounded by limited access to acute stroke care, rehabilitation services and long-term support for survivors.^32,33,34^ Stroke care in Cameroon is far below the recommended global standard. The critical shortage of stroke specialists and lack of diagnostics and therapeutics in these settings often prevent optimal and comprehensive acute and post-stroke care as well as community reintegration. With suboptimal care, patients may not fully recover from their deficits. These can contribute to the development of depression and other mental health comorbidities. Furthermore, social determinants of health such as poverty, limited social support networks and stroke-related stigma may also explain the high prevalence of depression in our patients. In Cameroon, only one stroke support group^35^ exists and is not very accessible to every stroke survivor. The disruption in social and economic roles in life after stroke can further compound these challenges. Stroke-related stigma, which is often fuelled by myths and misconceptions stemming from low levels of public awareness can result in the isolation of stroke patients, leading to increased vulnerability to PSD.^36^ In line with other studies, a lower Barthel index score indicating greater disability was associated with higher rates of PSD.^11,12,37^ Higher disability entails higher functional impairment, and this can profoundly impact their quality of life resulting in low self-esteem and decreased ability to engage in meaningful activities. These can heighten the risk of PSD. With greater disability, patients often require more intensive and prolonged rehabilitation and psychosocial support, which may not always be possible in Cameroon because of the limited availability of rehabilitation services and support groups. Fatigue or loss of energy is a common feature of depression and one of the criteria for the diagnosis of depression.^38^ Post-stroke fatigue may be the result of PSD or stroke-related disability. In either case, post-stroke fatigue can negatively affect self-motivation and interest in activities that can improve mental health in stroke survivors such as exercise and social participation, ultimately contributing to social withdrawal.^39^

Participants who recently had a stroke had higher PSD scores. This could be explained by the fact that those who experienced their stroke more recently may still be adjusting to the physical and emotional changes and thus be more vulnerable to developing depressive symptoms.^40^ Also, participants with a longer duration have had more time to develop coping skills, adapt and recover reducing their risk of depression.^40^ Moreover, over time, participants with a longer duration of stroke may seek and receive more support, which could also mitigate depressive symptoms. Furthermore, early depressive symptoms may resolve over time as the individual adapts to post-stroke challenges. Overall, the association of recent stroke with higher PSD scores underscores the need for close monitoring and proactive psychological support, especially in the initial months following a stroke.^16^ At the early stages of stroke recovery, there is a critical need to proactively screen for and manage depressive symptoms as part of a comprehensive rehabilitation programme.^16,40^

Social support and connections are well-recognised mitigating factors against mental health disorders like depression.^41^ In sub-Saharan African countries such as Cameroon, families are often under socioeconomic pressure. Caregivers of stroke survivors may prioritise working extra time to earn more money to cover medical expenses than spending extra time to give adequate moral, emotional and physical support to patients. Also, caregivers may see stroke survivors as an extra burden and may not fully engage in providing social support. This lack of a reliable social safety net could result in hopelessness experienced by stroke survivors. Community support programmes for stroke survivors in African countries like Cameroon are quasi-existent.^42^ A lack of understanding of stroke may cause communities to stigmatise stroke survivors and socially exclude them instead of supporting recovery.^36,43,44^

Our findings also suggest that stroke survivors who are divorced are more likely to experience PSD compared to those who are married. In a similar study conducted among stroke survivors in Nigeria, stroke survivors who were widowed or divorced had the highest scores on depression and suicidal ideations.^45^ Being divorced has been shown to negatively impact quality of life, and is a negative predictor of poor mental health.^46^ Evidence from research shows that married people enjoy better mental health than those who are divorced and marriage increases psychological, social and economic resources.^47,48^ Using systematic screening, we found a high prevalence of PSD. Our findings underscore the need for systematic screening and the integration of mental health support into routine stroke care. A ‘watchful waiting’ approach where screening is only done when a patient or their caregiver complaints will lead to late diagnosis and worse stroke outcomes. Moreover, people with depression are less likely to seek medical help,^49,50^ especially in the African setting where being depressed may be seen as a sign of weakness. In our study cohort, none of the patients complained of depressive symptoms or had previously consulted for depression.

## Conclusion

In this study, we found a high prevalence of PSD using a systematic screening approach. Implementing systematic screening for PSD in all stroke patients can lead to early diagnosis and, consequently, early initiation of treatment. Clinicians should not wait for patients or caregivers to report symptoms of PSD before conducting screenings.

### Limitations

The cross-sectional design precluded the determination of causal relationships. Additionally, the use of screening instruments instead of clinical interviews to assess depression may have led to an overestimation of prevalence. Our study population was limited to a hospital setting, and we recruited patients from a single site using a consecutive sampling method. Moreover, significantly impaired stroke survivors were excluded. These limitations affect the generalisability of our findings. Further studies are needed to explore the complex interplay of biopsychosocial factors that may contribute to PSD among stroke survivors in Cameroon across diverse settings.
